# Inhibition of PERK signaling suppresses tumor progression and blocks GP73-GRP78-dependent stromal activation in hepatocellular carcinoma

**DOI:** 10.1016/j.neo.2026.101329

**Published:** 2026-06-16

**Authors:** Jaafar Khaled, Maria Kopsida, Tania Payo Serafín, Sofi Sennefelt Nyman, Fredrik Rorsman, Charlotte Ebeling Barbier, Hans Lennernäs, Markus Sjöblom, Femke Heindryckx

**Affiliations:** a Department of Medical Cell Biology, Uppsala University, Husargatan 3, Uppsala 75123, Sweden; b Institute of Biomedicine (IBIOMED), Universidad de León, León, Spain; c Department of Surgical Sciences, Section of Radiology, Uppsala University, Uppsala, Sweden; d Department of Medical Sciences, Section of Gastroenterology and Hepatology, Uppsala University, Uppsala, Sweden; e Translational Drug Development and Discovery, Department of Pharmaceutical Biosciences, Uppsala University, Uppsala, Sweden

**Keywords:** Hepatocellular carcinoma, Endoplasmic reticulum stress, PERK pathway, GP73, Tumor-stromal interactions

## Abstract

Endoplasmic reticulum (ER) stress contributes to hepatocellular carcinoma (HCC) progression and promotes the development of a pro-tumorigenic microenvironment. Here, we demonstrate that selective inhibition of the ER-stress sensor PERK using AMG-PERK substantially restrains tumor development when administered during early carcinogenesis in a chemically induced HCC model. PERK inhibition reduced tumor burden, proliferation, and cell viability *in vivo*, and impaired the growth of HCC cells and patient-derived organoids *in vitro*. In parallel, AMG-PERK markedly reduced stromal activation, fibrosis, and inflammatory signaling within the tumor microenvironment. Mechanistic analyses indicated that ER-stress enhances tumor-stromal communication in part through increased secretion of the glycoprotein GP73, which can activate hepatic stellate cells via GRP78-dependent signaling. Blocking PERK or using GRP78-targeting antibodies reduced stellate cell activation and fibrogenic responses. Single-cell RNA sequencing and patient biopsies showed that PERK/EIF2AK3 and GP73/GOLM1 are upregulated in malignant hepatocytes and associated with poor clinical outcomes. Transcriptomic profiling further revealed that ER-stress drives oncogenic programs, including MYC signaling, epithelial-to-mesenchymal transition, and inflammatory pathway activation, all of which were affected by pharmacological PERK inhibition. Together, these findings identify PERK signaling as a potential driver of malignant progression and microenvironmental remodeling in HCC and establish PERK inhibition as a promising therapeutic strategy to target both tumor cells and their stromal interactions during the initial stages of hepatocarcinogenesis.

## Graphical abstract

**Figure:**
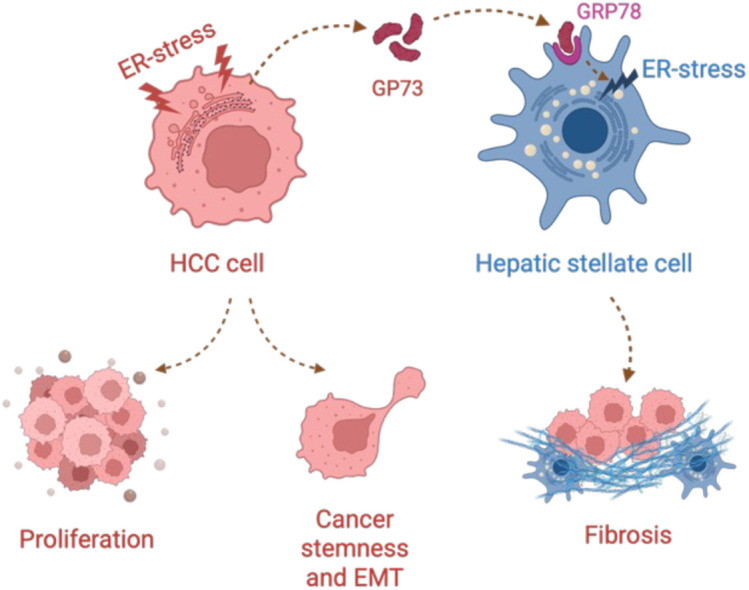

## Introduction

Hepatocellular carcinoma (HCC) is the most common primary liver cancer and a major cause of cancer-related mortality worldwide [1,2]. Most cases arise in the context of chronic liver injury, including viral hepatitis, alcohol-associated liver disease, and metabolic dysfunction-associated steatotic liver disease (MASLD) [1,3,4]. These conditions cause persistent hepatocellular damage and inflammation, which gradually evolve into fibrosis and ultimately cirrhosis, the major underlying risk factor for HCC [5,6]. Because cirrhosis represents an irreversible state with a substantially elevated lifetime risk of malignant transformation, there is a clinical need for preventive strategies that can intervene early in the carcinogenic process and limit progression from cirrhosis towards tumor formation [4,[6], [7], [8]].

During early carcinogenesis, hepatocytes acquire genomic instability, while the surrounding tumor microenvironment (TME) becomes increasingly fibrotic and immunomodulatory [8,9]. During this early stage, hepatocytes display molecular and genetic aberrations, including chromosomal gains, point mutations, telomere erosion, as well as telomerase reactivation and degradation [10,11]. Beyond these changes in hepatocytes, stromal cells, particularly hepatic stellate cells (HSCs) play a central role in the development and progression of HCC. Once activated, HSCs secrete extracellular matrix proteins, growth factors, and cytokines that perpetuate fibrosis and promote epithelial-mesenchymal transition (EMT), tumor growth, and metastatic potential [12,13]. Increasing evidence suggests that malignant hepatocytes release soluble factors that directly activate HSCs, creating a reciprocal feedback loop in which stromal activation reinforces tumor progression [14,15]. However, the molecular signals that mediate this tumor-stroma communication remain poorly defined.

One regulator of tumor-stroma dynamics is the endoplasmic reticulum (ER) stress signaling pathway [[16], [17], [18]]. Under normal conditions, the ER is responsible for the synthesis and folding of membrane and secretory proteins to maintain homeostasis [19]. However, when ER function is overloaded, due to excessive protein synthesis demands or pathological insults, misfolded or unfolded proteins accumulate in the ER lumen, triggering the unfolded protein response (UPR) to restore homeostasis or induce apoptosis [[19], [20], [21], [22]]. UPR signaling is mediated by three transmembrane pathways: protein kinase RNA-like ER kinase (PERK), inositol-requiring enzyme 1 (IRE1α), and activating transcription factor 6 (ATF6) [20,23]. PERK activation, in particular, has been shown to promote tumor survival [24,25], metabolic adaptation, and immune evasion [26,27], yet its role in coordinating tumor–stromal interactions during HCC development and progression is not well understood.

Recent studies have identified ER-stress as an important modulator of the tumor microenvironment [26,27], as UPR activation in tumor cells supports their survival but can also transmit stress signals to adjacent stromal and immune cells, including fibroblasts, endothelial cells, and macrophages [28]. One mechanism involves the ER-stress-induced secretion of factors such as Golgi Protein 73 (GP73), which can bind to the extracellular GRP78 receptor on macrophages and promote a tumor-associated phenotype [29]. Whether similar ER-stress-dependent signals contribute to communication with other stromal components, particularly HSCs that are central to HCC development and progression, remains unclear.

To address this gap, we investigated the role of PERK signaling during early hepatocarcinogenesis using AMG-PERK44, a highly selective type II PERK inhibitor [30]. Using a chemically induced HCC mouse model, human HCC cell lines, and patient-derived organoids, we examined how early pharmacological inhibition of PERK influences tumor initiation, progression, and TME remodeling. We further explored ER-stress-dependent mechanisms of tumor-stromal communication, including the contribution of the secreted glycoprotein GP73 to hepatic stellate cell activation. Our study reveals that early PERK inhibition slows down tumor development, suppresses stromal activation, reduces fibrosis, and reverses pro-tumorigenic transcriptional programs. These findings identify PERK signaling as an important driver of malignant and microenvironmental changes in early HCC and highlight the potential of PERK-targeted therapy as an intervention strategy during the initial stages of hepatocarcinogenesis, for instance in patients with liver cirrhosis.

## Methods

### Mouse model

A chemically induced HCC mouse model was used, as previously described [31,32]. 5-week-old male sv129 mice were intraperitoneally injected with 35 mg/kg body weight N-nitrosodiethylamine (DEN) in saline every other week for 15 weeks (N0258-1G, Sigma-Aldrich, Darmstadt, Germany), except for the healthy group, which got saline injections in the same volume. On week 16, mice were randomly allocated to different treatment groups, with nine mice in each group. Treatment groups received intraperitoneal injections twice per week with 10 μg/g bodyweight of AMG-PERK 44 (SML3049, Sigma-Aldrich, Darmstadt, Germany) in saline or control for 11 weeks. After 25 weeks, mice were euthanized for sample collection. All procedures were approved by the Uppsala Ethical Committee for animal experimentation (DNR 5.8.18-0089/2020) and followed RESIST guidelines.

Liver tissue was prepared for paraffin embedding by first rinsing half of the left liver lobe in ice-cold saline solution, before fixation in 4% paraformaldehyde for 24 hours. For mRNA-analyses, liver tissues were submerged in RNA-later solution (Sigma-Aldrich, Darmstadt, Germany) and incubated on ice for half an hour, before being snap frozen on dry ice and stored at −80°C. For protein analyses, tissues from the right liver lobe were snap frozen on dry ice and stored at −80°C.

### Study compounds

AMG-PERK44 is a small molecule (molecular mass = 561.1 g/mol) that is a potent and highly selective PERK inhibitor with an IC50 of 6 nM [33]. In mouse, it has a half-life of approximately 3 hours, total clearance 0.25 l/h/kg and a volume of distribution (SS) of 1.6 l/kg. When assuming a high plasma protein binding of AMG-PERK44 (∼ 90%) and a bioavailability at 100% following intraperitoneal administration, the mean plasma exposure of free drug at steady-state is predicted to be at least 10 times higher than the reported IC50 value [30].

### Cell culture experiments

HepG2 (ATCC HB-8065), SNU449 (ATCC CRL-2234), and Huh7 (Tebu-bio JCRB0403-A) human HCC cell lines, as well as LX-2 human hepatic stellate cells, were cultured in a humidified incubator at 37 °C with 5% CO₂. HepG2 and Huh7 cells were maintained in high-glucose DMEM supplemented with GlutaMAX (ThermoFisher), 10% fetal bovine serum (FBS), and 1% antibiotic-antimycotic. SNU449 cells were cultured in RPMI 1640 with identical supplements. Cell line identities were verified by STR profiling (Eurofins Genomics), and all cell lines tested negative for misidentification in the Register of Misidentified Cell Lines.

For ER-stress induction and inhibition, Huh7, HepG2, SNU-449, and LX-2 cells were seeded and treated for 24 h with either vehicle (control), 1 μM thapsigargin (T9033 - 5MG, Sigma-Aldrich, Darmstadt, Germany)) [34,35], 5 nM AMG-PERK 44, or both. Following treatment, conditioned medium was collected for GP73 ELISA, and total RNA was extracted using standard protocols for subsequent qPCR analysis. Human GOLM1/GP73 (EH227RB, ThermoFisher, Stockholm, Sweden) levels were measured in medium using ELISA, according to the manufacturer's protocol. Results were calculated using the averages from 3 biological replicates and 3 technical replicates.

LX-2 cells were treated with 5 µg/mL recombinant human GP73 (P1316, FineTest, Wuhan National Bio-industry Base, Biolake, China) for 24 hours, with or without 5 nM AMG-PERK44. To test GRP78 involvement, LX-2 cells were pre-incubated with 5 µg/mL NT-GRP78/N-20 antibody (ab32618, Abcam, Cambridge, UK) for 2 hours, followed by 24 hours GP73 treatment. For conditioned medium experiments, Huh7, HepG2, and SNU-449 cells were treated with thapsigargin for 24 hours, and conditioned media were collected and centrifuged. LX-2 cells were then cultured for 24 hours in: (1) normal medium (control), (2) tumor-conditioned medium, (3) tumor medium with 5 µg/mL NT-GRP78 antibody, or (4) tumor medium with 5 nM AMG-PERK44. A Rabbit IgG istotype control (10500C, Invitrogen, ThermoFisher) was used as control.

### Histology and staining

Livers were fixed in 4% paraformaldehyde for 24 h, paraffin-embedded, sectioned at 8 µm, deparaffinized, and rehydrated. Hematoxylin-eosin staining followed standard protocols. Picrosirius Red (60 min) was used to visualize collagen. Collagen area (%) was quantified in a blinded manner in Fiji/ImageJ after color deconvolution of Sirius Red; nuclei were thresholded on the blue channel and collagen-positive area was expressed as percentage of tissue area per field.

For immunohistochemistry on liver tissue, paraffin sections were stained using an HRP–DAB kit (ab64261, Abcam). Antigen retrieval was performed in a decloaking chamber at 95°C (Diva Decloacker DV2004, Biocare). After protein block (30 min), sections were incubated overnight at 5°C with primary antibodies to Ki67 (ab197547, Abcam), PCNA (PA5-27214, ThermoFisher), αSMA (ab124964, Abcam), GP73/GOLPH2 (PA5-96624, ThermoFisher), PERK (PA5-82537, ThermoFisher) and Rabbit IgG isotype control (ref 10500C, Invitrogen, ThermoFisher). Images were acquired on a Nikon Eclipse TE2000-U microscope x10; DAB signal was quantified in Fiji/ImageJ by H-DAB color deconvolution using identical thresholds across groups.

For immunofluorescence on liver tissue, paraffin sections were processed as above and incubated overnight with β-catenin (ab2365, Abcam) or vimentin (ab92547, Abcam), followed by species-appropriate secondary antibodies (goat anti-mouse Alexa Fluor 488; goat anti-rabbit Alexa Fluor 555; ThermoFisher). Sections were counterstained with DAPI and mounted in Fluoromount-G. Confocal images (Zeiss LSM-700, 63 × /1.40 oil) were analyzed in Fiji/ImageJ; positive cells were quantified per mm² with uniform thresholds across samples. For immunofluorescence on cell lines, HepG2, Huh7, SNU449 and LX-2 cells were fixed in 4% paraformaldehyde for 30 min at 4°C, blocked, and stained overnight with ATF4 (PA5-27576, ThermoFisher), αSMA (ab124964, Abcam), EpCAM (ab71916, Abcam). Species-appropriate secondaries (goat anti-mouse Alexa Fluor 488; goat anti-rabbit Alexa Fluor 555; ThermoFisher) were applied for 1 h, nuclei were counterstained with DAPI/Hoechst, and coverslips were mounted in Fluoromount-G. Confocal images (Zeiss LSM-700; 20 × /63 ×) were quantified as mean fluorescence intensity or percentage of positive cells with fixed exposure and analysis parameters across conditions. For ER-Tracker staining, LX-2 cells were stained with ER-Tracker™ Green (E34251, ThermoFisher) and Hoechst 33342 (R37605, ThermoFisher) according to the manufacturer’s instructions. Confocal images (Zeiss LSM-700, 20 ×) were analyzed in Fiji/ImageJ; ER-Tracker signal was normalized to nuclear (DAPI/Hoechst) area for each field.

### Quantitative RT-PCR of mRNA

RNA isolation was performed on whole liver tissue and cell culture was carried out using the RNeasy Universal Mini Kit (74004, Qiagen, Sollentuna, Sweden) and the E.Z.N.A. Total RNA-Kit I (R6834-02, Omega Biotek, Inc., Norcross, Georgia, USA), respectively, in accordance with the manufacturer’s instructions. RNA concentration and purity were assessed with a Nanodrop spectrophotometer. For reverse transcription of the mRNA, the iScript cDNA-synthesis kit (1708891, Bio-Rad, Solna, Sweden) was used following the manufacturer’s protocol. Primers were designed with Primer Blast and obtained from ThermoFisher. Using Fast SYBR Green (Ref: 4385612, ThermoFisher Scientific), amplifications were performed in accordance with the manufacturer's guidelines. Gene expression levels were quantified with QuantStudio 5 (ThermoFisher Scientific). mRNA expression was normalized to GAPDH and/or 18S. Fold changes were determined based on average CT values of two technical duplicates for each sample using the delta-delta-CT method.

### Resazurin reduction (Alamar Blue) assay

Cell viability was conducted via a resazurin reduction assay. Cells were seeded onto Corning 96-well, flat and clear bottom (734-1610, Corning, VWR, Sweden) at a seeding density of 1.0 × 10^4^ cells for monocultures per well. Cells were then treated with AMG-PERK in a concentration range from 0,01 nM to 1000 nM for 24 hours. A 1% solution of resazurin sodium salt (R7017-1 G, Sigma-Aldrich, Darmstadt, Germany) was added to the cells at a 1/80 dilution, followed by a 24-hour incubation. Fluorescence was then measured using a Fluostar Omega plate reader with both excitation and emission filters at 540/35 nm and 590/20 nm wavelengths, respectively.

### Migration assay

Cell migration was assessed using a scratch wound healing assay. Briefly, SNU449-cells were cultured using RPMI 1640 medium supplemented with 1% antibiotic antimycotic solution and 10% FBS in six-well plates (Ref: 83.3920, SARSTEDT, Nümbrecht, Germany) at a seeding density of 2,5 × 105 for 72 hours. The cells were left to reach 100% confluency. Starvation medium of RPMI was then added for 4 hours, after which a scratch was created on the confluent cell layer, using a sterile 200 µL pipette tip. The cells were washed twice in phosphate buffered saline (PBS) to remove any debris. Thereafter, cells were treated with 5 nm AMG-PERK 44 in the presence of 30 nM of Mitomycin C (10107409001, Sigma-Aldrich, Darmstadt, Germany) to stop cell proliferation [36]. Cell migration into the scratch wound area was observed through light microscopy imaging. Photos were captured at 0, 24, 48 and 72 h after wounding. The gap distance was quantitatively measured using ImageJ.

### Enzyme-linked immune sorbent assay (E.L.I.S.A)

Human GOLM1/GP73 levels were measured in medium samples collected from cells using ELISA kit (EH227RB, ThermoFisher, Stockholm, Sweden), according to the manufacturer's protocol. Absorbance was measured at 450 nm using a Synergy H4 Multi-Mode Reader (BioTek Instruments). Results were calculated using the averages from 3 biological replicates and 3 technical replicates.

### Human liver cancer organoid isolation and culture

Needle biopsies were collected with ultrasound guidance from 5 non-tumoral liver tissues in 5 HCC patients (patient numbers #24; #25; #28; #29; #30) prior to transarterial chemoembolization (TACE) with idarubicin, as part of the TACTida clinical trial (Uppsala University; ethical approval Dnr 2021-01928, EUDRA CT 2021-001257-31) [37]. Written informed consent was obtained from all participants. Organoids were established from 5 mg of material using mechanical and enzymatic dissociation with the HepatiCult™ Organoid Kit (StemCell Technologies, #100-386) according to the manufacturer’s protocol [38]. Organoid cultures were initiated in HepatiCult™ Organoid Initiation Medium (#100-0384) and maintained in Organoid Growth Medium (#100-0385). Organoids were embedded in 15 µL droplets of Cultrex BME2 (Type 2, Reduced Growth Factor) and seeded at a density of 5  ×  10³ cells per droplet. Organoids were treated for 24 hours with AMG-PERK 44 and/or thapsigargin. Viability was assessed using CellTiter-Glo® 3D assay (G9683, Promega, Stockholm, Sweden), and luminescence was measured using a Synergy H4 Multi-Mode Reader (BioTek Instruments).

### SDS-PAGE and western blot

Western blot analyses were performed using whole liver tissue. Cell lysates were prepared and mixed with 2 × Laemmli buffer (S3401-10VL Sigma-Aldrich, Darmstadt, Germany). Samples were heated at 95°C for 5 min and separated by SDS-PAGE on precast 4-20% Mini-PROTEAN® TGX™ gels (456-1096 Bio-Rad, Solna, Sweden). Proteins were transferred onto Immobilon®-FL membranes (IPFL0010, Millipore, Solna, Sweden) and blocked in Intercept® TBS Blocking Buffer (927-60001, LI-COR, Bad Homburg, Germany) diluted 1:4 in PBS. For fluorescent detection of β-catenin (Ref: MA1-2001, ThermoFisher Scientific, Stockholm, Sweden) and Vimentin (ab92547, Abcam, Cambridge, UK), membranes were incubated with primary antibodies diluted in blocking buffer with 0.1% Tween-20, followed by IRDye 800CW Goat anti-Mouse IgG (ab216772, Abcam, Cambridge, UK) and Alexa Fluor 680 Goat anti-Rabbit IgG (Ref: A-21109, ThermoFisher Scientific, Stockholm, Sweden) secondary antibodies diluted in blocking buffer containing 0.1% Tween-20 and 0.01% SDS. Blots were scanned using an Odyssey scanner (LI-COR Biotechnology), and band intensities quantified with Odyssey 2.1 software. For chemiluminescent detection of β-actin (ab8227, Abcam, Cambridge, UK), membranes were incubated with goat anti-rabbit IgG HRP as secondary antibody, and signal was developed using clarity™ Western ECL (170-5060, Bio-Rad, Solna, Sweden) substrate Chemiluminescence was imaged using the Odyssey scanner and values normalized to β-actin signal [39].

### Single-cell RNA-seq data acquisition and analysis

Single-cell RNA sequencing (scRNA-seq) data were obtained from the Sequential NCI-CLARITY dataset retrieved from the Gene Expression Omnibus (GEO) database (Accession: GSE229772) [40]. This dataset profiles liver tumors at single-cell resolution, including both malignant HCC cells and non-malignant stromal populations. Analysis was conducted using the Single-Cell Atlas in Liver Cancer (scAtlasLC), a publicly available resource providing transcriptomic data in HCC. t-distributed stochastic neighbor embedding (t-SNE) plots were generated via scAtlasLC to visualize the clustering of malignant and non-malignant cells. Malignant cells were color-coded by patient of origin, while stromal non-malignant cells were annotated by cell type as defined in the dataset. Expression of GOLM1 (GP73) and EIF2AK3 (PERK) was mapped onto the t-SNE plots to assess their distribution across cellular subsets.

### Human protein atlas

Patient survival was correlated with the expression levels of GOLM1 (GP73) using RNA sequencing expression data from The Cancer Genome Atlas (TCGA), publicly available on the Human Protein Atlas [41,42]. GOLM1 levels across different HCC stages were evaluated using RNA sequencing data from 362 patients, who were then categorized into high- or low-expression groups based on fragments per kilobase of exon per million reads mapped (FPKM). The cut-off (FPKM) value from the Human Protein Atlas was adopted to classify the transcriptomic samples accordingly. All survival analyses were performed using GraphPad Prism 10.5. The optimal expression cut-off level was defined as the FPKM value that best separated patient groups with significantly different overall survival, as determined by the log-rank test with the lowest p-value. Kaplan–Meier survival curves were generated and visualized in Prism, and p-values of < 0.05 were considered statistically significant. Representative images of immunohistochemistry staining with antibodies against EIF2αK3 (ID: HPA015737) [43] and GOLM1 (ID: HPA010638) [44] in HCC biopsies were retrieved from the Human Protein Atlas [45].

### Gene set enrichment analysis (GSEA)

Publicly available RNA sequencing data comprising expression levels of 19,891 genes were retrieved from the GEO database (Accession: GSE208391) [46,47]. The dataset consists of HepG2 cells treated for 24 hours with tunicamycin, an ER-stress inducer. The RNA-Seq dataset was generated using the Illumina HiSeq 2000 platform, with paired-end sequencing, Quality Control and Preprocessing. Prior to analysis, FastQC (Galaxy) was used to assess the quality of raw sequencing reads. Adapter contamination, per-base sequence quality, GC content, and sequence duplication levels were checked. As all sequences passed quality control, trimming was not required. The paired-end RNA-Seq reads were aligned to the GRCh38 (hg38) reference genome using HISAT2 (Galaxy). Default parameters were used, and alignment was performed separately for each sample. Gene expression levels were quantified from the aligned RNA-Seq data using FeatureCounts (Galaxy).

The dataset was divided into control (untreated HepG2 cells) and tunicamycin-treated groups. Differential expression analysis was conducted using DESeq2, and genes were ranked by their log₂ fold change for subsequent enrichment analyses. Gene Set Enrichment Analysis (GSEA) was performed using the clusterProfiler R package (v4.6.0) to identify pathways associated with expression changes in our dataset. For GSEA, we used the Hallmark gene sets (H) from the Molecular Signatures Database (MSigDB v2023.2) obtained via the msigdbr R package. Gene symbols were mapped to Entrez gene identifiers using the org.Hs.eg.db annotation database. Genes were filtered for complete mapping and ranked, and GSEA was run using the GSEA() function with 10,000 permutations and a p-value cutoff of 0.05. KEGG pathway enrichment was performed using the enrichKEGG() function from clusterProfiler, using the list of significantly differentially expressed genes (adjusted p-value < 0.05 and |log₂FC| > 1). Genes were converted to Entrez identifiers before enrichment. Enriched pathways were visualized with dotplot() and barplot() functions from the enrichplot package. Gene Ontology (GO) enrichment analysis was performed for Biological Process (BP) terms using the enrichGO()function in clusterProfiler. Significant DEGs were annotated using Entrez IDs, and enrichment was computed with a p-value and q-value cutoff of 0.05. Enrichment results were visualized using dot plots and bar plots generated via enrichplot. All analyses were conducted in R version 4.4.0, and visualizations were generated using ggplot2 and enrichplot.

### Transcriptomics

Transcriptomic datasets involving genetic PERK depletion were analyzed to investigate whether the effects observed following pharmacological PERK inhibition were mechanistically linked to PERK signaling. The following GEO datasets were included: liver-specific PERK knockout mice (LsPERK-KO; accession: GSE29929 [48]), PERK knock out LX2 stellate (GSE292002) [49], and PERK-deficient bone marrow-derived macrophages (Accession: GSE165836) [26]. For microarray data (Accession: GSE29929), expression values were log2 transformed and quantile normalized using the limma package in R. RNA-sequencing datasets (GSE292002 and GSE165836) were analyzed using TPM or FPKM values obtained from the processed supplementary files, followed by log2(TPM+1) or log2(FPKM+1) transformation. Gene expression levels of inflammatory, fibrotic, and ER-stress-related markers, including Il6, Tnf, Arg1, Col1a1, Acta2, Tgfb1, Golm1, and Hspa5, were extracted and compared between control and PERK-deficient conditions.

### Statistical analysis

Data are presented as mean ± standard deviation (SD). Statistical significance was determined using an unpaired, two-tailed Student’s T-test or one-way analysis of variance (ANOVA), followed by Tukey’s multiple comparison test. A P-value of <0.05 was considered statistically significant. GraphPad Prism 9 was used to perform statistical analyses and generate graphs. *In vitro* experiments were conducted with a minimum of three biological replicates, defined as parallel measurements of biologically distinct samples obtained from independent experiments. Technical replicates refer to multiple loadings of the same sample within the final assay. *In vivo* experiments were performed on at least five independent animals. Outliers were included in the analysis unless deemed to result from technical errors, in which case the experiment was repeated.

## Results

### Early pharmacologic PERK inhibition reduces tumor burden and proliferation in vivo and decreases cell viability in vitro

**Fig. 1:**
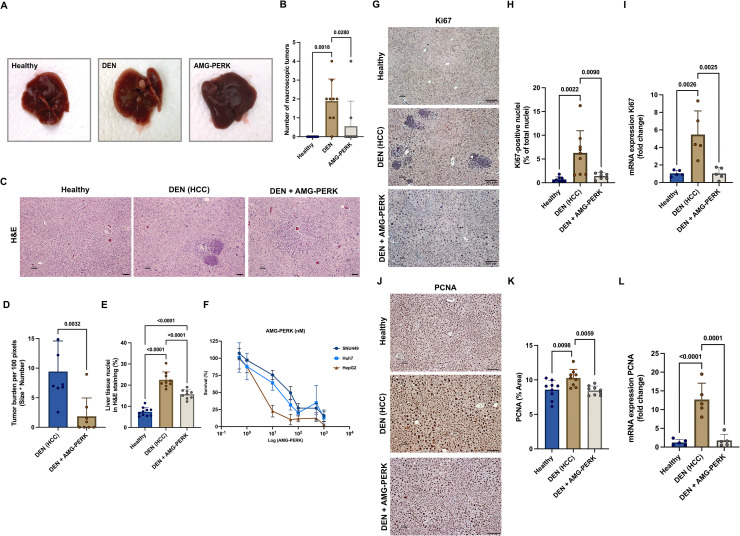
Treatment with AMG-PERK reduces tumor burden, proliferation, and viability. (A) Representative images of mouse livers. (B) Quantification of macroscopic tumors per liver. (C) Representative H&E-stained liver sections. Scale bar: 100 μm. (D) Quantification of tumor burden on H&E-stained slides. (E) Percentage of nuclei in liver tissue. (F) Cell viability in human HCC cell lines (HepG2, Huh7, SNU449) treated with AMG-PERK. (G, J) Representative images of Ki67 and PCNA immunohistochemical staining. Scale bar: 200 μm. (H) Quantification of Ki67-positive nuclei as a percentage of total nuclei. (I) mRNA expression of Ki67 (K) Quantification of percentage PCNA-positive staining per area (L) mRNA expression of PCNA. Bars represent mean with ± SD. N = 8 mice per group for tumor burden; N = 10 (Healthy) and 9 (DEN, DEN + AMG-PERK) for H&E; N = 9 mice per group for PCNA; N = 8 (Healthy), 9 (DEN), and 7 (DEN + AMG-PERK) for Ki67. qPCR: N = 5 mice/group.

To assess the impact of PERK inhibition during early hepatocarcinogenesis, mice were injected with DEN for 15 weeks and subsequently treated with the selective PERK inhibitor AMG-PERK or vehicle control for an additional 10 weeks. At endpoint, DEN-treated mice displayed multiple macroscopic tumors, whereas AMG-PERK–treated mice exhibited markedly fewer visible lesions (Fig. 1A). Quantification confirmed a significant reduction (P = 0.0280) in tumor number following AMG-PERK treatment (Fig. 1B). Histological analysis of liver tissue using H&E staining showed significantly lower tumor burden (P = 0.0032) in AMG-PERK-treated mice compared to untreated DEN HCC mice (Fig. 1C, D). This was further supported by increased nuclear density in HCC livers, consistent with highly proliferative tumor regions [50], while AMG-PERK treatment led to a considerable reduction in nuclear density (Fig. 1E). *In vitro*, AMG-PERK treatment led to a dose-dependent reduction in cell viability across three HCC cell lines (Fig. 1F). Immunohistochemical staining for proliferation markers PCNA and Ki67 showed a high percentage of positive cells in DEN-induced HCC livers, and a clear reduction following AMG-PERK treatment (Fig. 1G, H, J-K, Supplementary Fig. S1). Quantitative PCR analysis supported these findings, showing elevated mRNA-expression levels of ki67 and PCNA in DEN livers that were significantly reduced (ki67 P = 0.0025; PCNA P = 0.0001) in the AMG-PERK group (Fig. 1I, L).

Effective PERK pathway inhibition in vivo was confirmed by reduced expression of ER-stress markers in AMG-PERK-treated livers, as shown by qPCR, immunohistochemistry, and immunofluorescence (Supplementary Fig. S2). These findings are consistent with the predicted steady-state plasma exposure of AMG-PERK44, which is sufficient to inhibit PERK activity in mice [30]. In vitro, AMG-PERK also suppressed ER-stress signaling across multiple HCC cell lines (Supplementary Fig. S3), with effects observed at concentrations within the range of estimated free drug levels. As determined by pharmacokinetic parameters and high plasma protein binding (∼90%), the estimated free drug concentration of AMG-PERK44 in mice lies at approximately 60-80 nM, corresponding closely to the concentrations used in our models. qPCR analysis also confirmed transcriptional suppression of ER-stress signaling the different cell lines (Supplementary Fig. S3). The *in vitro* effects were observed at 5 nM, which is lower than the expected *in vivo* exposure.

### PERK inhibition remodels the tumor microenvironment in vivo and affects viability in patient-derived liver organoids

**Fig. 2:**
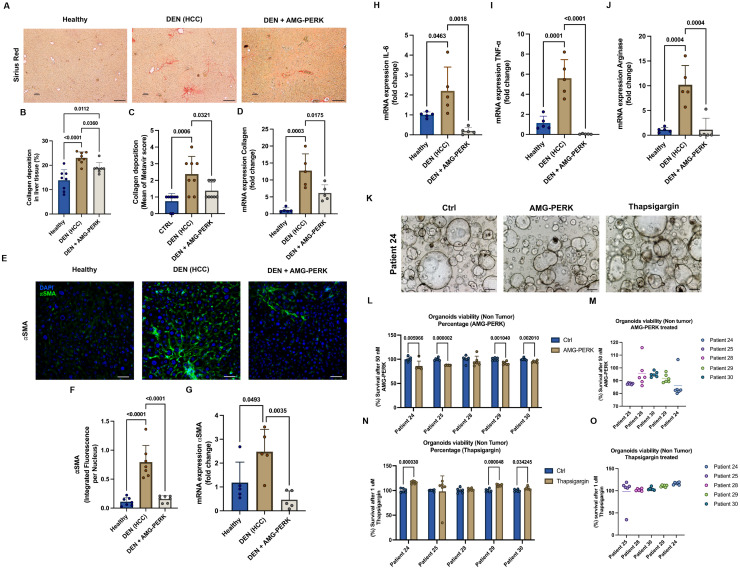
Treatment with AMG-PERK reduces fibrosis and inflammation. (A) Representative Sirius Red–stained liver sections. Scale bar: 200 μm. (B) Quantification of collagen deposition. (C) Metavir scoring of fibrosis. (D) mRNA expression of Col1a1. (E) Representative αSMA-antibody-stained immunofluorescence images. Scale bar: 50 μm. (F) Quantification of αSMA-positive staining normalized per nuclear staining. (G-J) mRNA expression of αSMA, IL-6, TNF-α and Arginase. (K) Representative bright-field images of patient-derived liver organoids treated with 50 nM AMG-PERK or 1 µM thapsigargin for 24 h. Scale bar: 200 μm. (L-M) Viability and inter-patient variability after AMG-PERK exposure. (N—O) Viability and inter-patient variability after thapsigargin exposure. Organoid data represent mean ± SD of three independent experiments with technical duplicates. Bars represent mean with ± SD. N = 8 mice per group for Sirius Red; N = 7 mice per group for αSMA; qPCR: N = 5 mice/group.

To examine how ER-stress inhibition influences TME, we first assessed fibrosis and inflammation in DEN-induced HCC livers. Sirius Red staining showed moderate collagen deposition in DEN-mice, which was reduced in AMG-PERK-treated animals (Fig. 2A, B). This was supported by lower Metavir scores in the treatment group (Fig. 2C) and significantly decreased (P = 0.0175) collagen mRNA expression (Fig. 2D). Additionally, expression of the stellate cell activation marker alpha smooth muscle actin (αSMA) was reduced at both the mRNA and protein levels in AMG-PERK-treated mice, as confirmed by qPCR and immunofluorescence staining (Fig. 2E–G, Supplementary Fig. S4), indicating attenuation of fibrosis through ER-stress inhibition. Expression levels of pro-inflammatory cytokines TNF-α and IL-6, as well as the immune marker arginase, were elevated in the livers of DEN-mice, but were substantially decreased in the treatment group (Fig. 2H–J), suggesting that ER-stress inhibition mitigates inflammatory signaling and affects the pro-tumorigenic tumor microenvironment. To evaluate whether ER-stress modulation affects the TME in patients, we generated patient-derived liver organoids from non-tumoral biopsies of HCC patients. Treatment with 50 nM AMG-PERK (comparable to estimated free drug levels *in vivo*) reduced viability in most organoid lines, although with notable inter-patient variability (Fig. 2K–M). Conversely, exposure to the ER-stress inducer thapsigargin (1 µM) increased viability across most organoids, again with some variation between patients (Fig. 2N, O). While the magnitude of these effects was modest, the responses were substantial and consistent in direction, supporting a patient-specific relationship between ER-stress modulation and cellular response. Importantly, the agreement between active drug concentrations in organoids and predicted *in vivo* exposure reinforces the translational relevance of these findings.

To further investigate whether the effects observed after pharmacological PERK inhibition were mechanistically linked to PERK signaling itself, we analyzed three publicly available datasets involving genetic PERK depletion in different cell types and ER-stress contexts (GSE29929, GSE292002 and GSE165836 [26,48,49]). In liver-specific PERK knockout mice (LsPERK-KO), PERK deletion alone did not markedly alter basal expression of inflammatory or fibrotic markers, including *Tnf, Arg1, Col1a1, Acta2* (αSMA) and *Tgfb1*, compared to WT controls (Supplementary Fig. S5-A). However, following induction of ER-stress using tunicamycin, *Il6* expression was significantly reduced in LsPERK-KO mice relative to tunicamycin-treated WT animals (Supplementary Fig. S5-B). Similarly, analysis of PERK-deficient LX2 stellate cells exposed to palmitate-induced lipotoxic stress (GSE292002) demonstrated reduced expression of *Il6* and *Tgfb1*, supporting a role for PERK signaling in inflammatory and fibrogenic activation under stress conditions (Supplementary Fig. S5-C). In contrast, PERK deletion in bone marrow-derived macrophages (LysMCre x Eif2ak3fl/fl; GSE165836) had limited effects on *Arg1, Tgfb1* and *Tnf* expression, while *Il6* expression was largely absent in this dataset (Supplementary Fig. S5-D).

### Single-cell profiling of GOLM1/EIF2AK3 and pharmacologic inhibition of PERK signaling reveal ER-stress–responsive regulation of GP73 in HCC

**Fig. 3:**
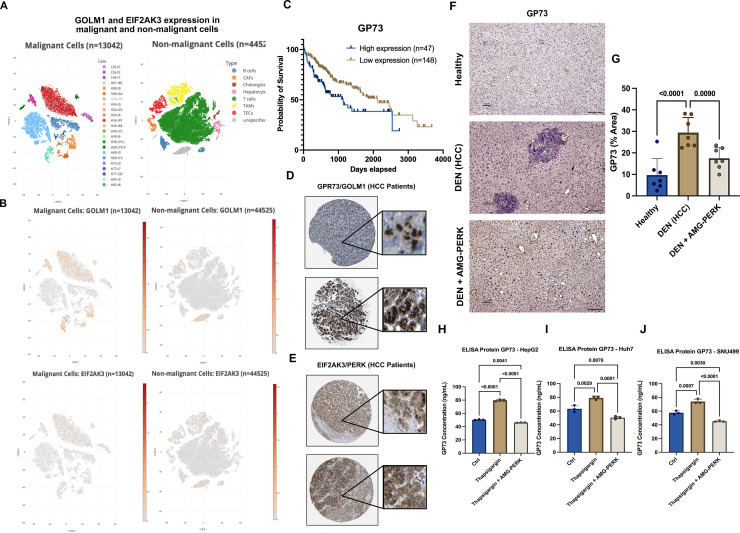
Integration of single-cell expression data and PERK pathway inhibition demonstrates ER-stress–dependent regulation of GP73 in hepatocellular carcinoma. (A) t-SNE plots depicting clustering of malignant (left) and non-malignant (right) cells, color-coded by patient (malignant) or cell type (stroma). (B) Expression of GOLM1 and EIF2αK3 mapped onto t-SNE plots, derived from Sequential NCI-CLARITY (GSE229772) and analysed using the Single-Cell Atlas in liver cancer (scAtlasLC) (C) Kaplan-Meier survival curves of HCC patients with low and high expression of GP73, derived from the Cancer Genome Atlas (TCGA). (D-E) Immunohistochemistry images of EIF2αK3 and GOLM1 expression levels in HCC biopsies, derived from the Human Protein Atlas, proteinatlas.org. (F) Representative GP73-antibody-stained immunohistochemistry images from mouse livers. Scale bar: 200 μm. (G) Quantification of the GP73-positive area. (H-J) Quantification of GP73 secretion in HepG2, Huh7, and SNU449 cells treated with thapsigargin alone or in combination with AMG-PERK. Bars represent mean with ± SD. N = 7 mice per group for GP73 IHC.

Given the effects of PERK inhibition on tumor progression and the TME, we next examined potential ER-stress–responsive factors that may contribute to tumor–microenvironment interactions. Golgi protein 73 (GP73, encoded by GOLM1) is a secreted glycoprotein previously linked to HCC progression and clinical outcome, and reported to be stress-responsive in liver disease [51,52]. To characterize GOLM1/GP73 and EIF2AK3/PERK expression across cellular compartments, we analyzed single-cell RNA-sequencing data from malignant and non-malignant liver cells in the NCI-CLARITY dataset (GSE229772) [53]. t-SNE clustering was used to visualize malignant cells (color-coded by patient) and non-malignant cells, including T cells, macrophages, endothelial cells, and hepatocytes (Fig. 3A). Both GOLM1 and EIF2AK3 were increased in malignant cells, with slightly lower expression observed in the surrounding stroma (Fig. 3B). Among non-malignant cells, GOLM1 expression was primarily restricted to cancer-associated fibroblasts (CAFs), tumor endothelial cells (TECs), and cholangiocytes. EIF2AK3, in contrast, showed broader expression across stromal populations, albeit fairly low. To evaluate the clinical relevance, we performed survival analysis using TCGA data, which revealed that high GOLM1/GP73 expression correlates with poorer overall survival in HCC patients (Fig. 3C). Immunohistochemical staining from the Human Protein Atlas [45] confirmed increased GOLM1 and EIF2AK3 expression in HCC biopsies (Fig. 3D, E).

We then assessed whether ER-stress modulation influences GP73 expression and secretion. In DEN-induced HCC mice, immunohistochemistry revealed increased GP73-positive area in the untreated group, whereas AMG-PERK treatment reduced GP73 expression in the liver (Fig. 3F, G). *In vitro,* induction of ER-stress with thapsigargin increased GP73 secretion across three HCC cell lines (HepG2, Huh7, and SNU449) (Fig. 3H–J). Notably, co-treatment with AMG-PERK prevented the thapsigargin-induced increase in GP73 secretion, suggesting that GP73 expression and release are responsive to PERK-dependent ER-stress signaling (Fig. 3H–J). ER-stress pathway modulation was confirmed, as Thapsigargin activated classical ER-stress markers (Supplementary Fig. S2), and these markers were suppressed by AMG-PERK (Supplementary Figs. S1, S2).

### Tumor-conditioned signals and GP73 stimulate ER-stress and fibrotic responses in hepatic stellate cells

**Fig. 4:**
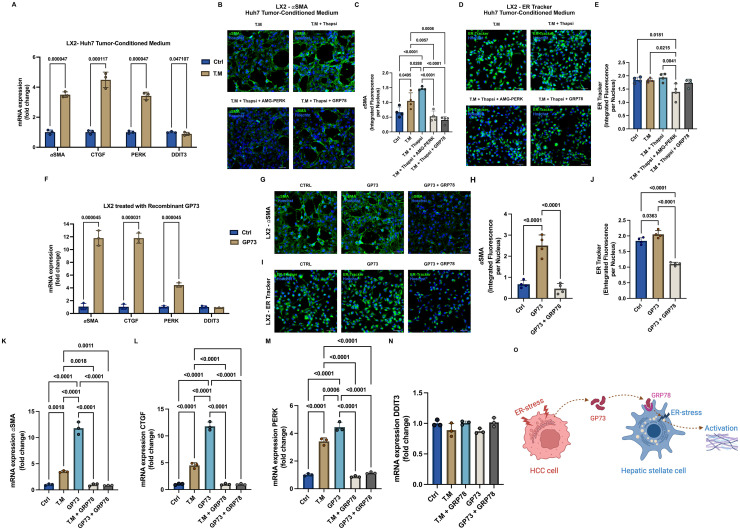
PERK inhibition reduces GP73-associated ER-stress and fibrotic responses in hepatic stellate cells. (A) mRNA expression of αSMA, CTGF, PERK, and DDIT3 in LX-2 cells treated with tumor-conditioned medium (T.M.) derived from Huh7 cells (B) Representative αSMA immunofluorescence images of LX-2 cells treated with T.M., T.M. + thapsigargin (Thapsi), T.M. + Thapsi + AMG-PERK, T.M. + Thapsi + a blocking antibody against the extracellular binding site of the surface receptor GRP78 (C) Quantification of αSMA staining normalized to nuclear staining (DAPI). (D) Representative ER-Tracker staining under the above-mentioned conditions. (E) Quantification of ER-Tracker signal normalized to nuclear staining (DAPI). (F) mRNA expression of αSMA, CTGF, PERK, and DDIT3 in LX2 cells treated with recombinant GP73. (G-J) Representative images and quantification of αSMA and ER-Tracker staining in LX-2 cells treated with GP73 alone or in combination with a blocking antibody against the extracellular binding site of the surface receptor GRP78. (K-N) qPCR analysis of αSMA, CTGF, PERK, and DDIT3 across control, TM, GP73, TM + GRP78 blockade and GP73 + GRP78 blockade. (O) Schematic representation of a proposed model (created with BioRender.com). Bars represent mean with ± SD. N = 3 biological replicates for qPCR; N = 4 biological replicates for staining.

To examine how tumor-derived signals influence HSC activation, we first exposed LX-2 cells to tumor-conditioned medium (TM) collected from Huh7 cells. TM increased the mRNA expression of αSMA, CTGF, and PERK, whereas DDIT3 remained unchanged (Fig. 4A). Consistent with these transcriptomic changes, TM increased αSMA-positive staining (Fig. 4B and C). This indicates that soluble factors released by tumor cells activate HSCs. When ER-stress was further enhanced by thapsigargin (Thapsi), αSMA staining increased even more (Fig. 4B, C). This was reduced to near-baseline levels when cells were co-treated with AMG-PERK or with a blocking antibody against the extracellular domain of surface GRP78 [54] (Fig. 4B, C). ER-Tracker staining showed a different pattern: TM or TM + Thapsi did not substantially increase ER-Tracker signal, but TM + Thapsi + AMG-PERK resulted in a decrease compared to TM-treated cells, whereas no reduction was observed with GRP78 blockade (Fig. 4D, E).

To assess whether GP73 would give a similar effect as TM, LX2 cells were treated with recombinant GP73. Treatment with recombinant GP73 significantly increased αSMA (P = 0.000134), CTGF (P = 0.000030), and PERK (P = 0.000096) mRNA expression, while DDIT3 remained unchanged (Fig. 4F), similar to the effects of TM (Fig. 4A). GP73 also increased αSMA-positive cells and slightly increased ER-Tracker signal (Fig. 4G–J). Co-treatment with the GRP78-blocking antibody reduced GP73-induced αSMA staining and ER-Tracker signal (Fig. 4G–J). To compare TM- and GP73-induced responses directly, we analyzed mRNA expression of αSMA, CTGF, PERK, and DDIT3 across multiple conditions (control, TM, GP73, TM + GRP78 blockade, GP73 + GRP78 blockade). TM increased αSMA, CTGF, and PERK (Fig. 4K–N), while GP73 induced even stronger upregulation of these genes. In both cases, GRP78 blockade substantially reduced the fibrotic and ER-stress-associated gene expression patterns (Fig. 4K–N). DDIT3 remained unchanged across all conditions. Together, these data show that tumor-conditioned signals, such as GP73, can activate stellate cells at both transcriptional and protein levels, that these responses likely involve surface GRP78 and that they are modulated by PERK signaling (Fig. 4O).

To control for nonspecific antibody effects and validate the specificity of GRP78 antibody-mediated responses, an isotype-matched IgG control antibody was included in parallel experiments. No significant differences in PERK, DDIT3, αSMA, or CTGF mRNA expression were observed between T.M. and T.M. + isotype IgG control (Supplementary Fig. S6 A–D) or between GP73 and GP73 + isotype IgG control conditions (Supplementary Fig. S6 E–H). Additionally, isotype IgG control staining of DEN-induced HCC mouse liver sections showed no immunohistochemical signal (Supplementary Fig. S3 I).

### ER-stress promotes oncogenic and EMT-related transcriptional programs that are attenuated by pharmacologic PERK inhibition

**Fig. 5:**
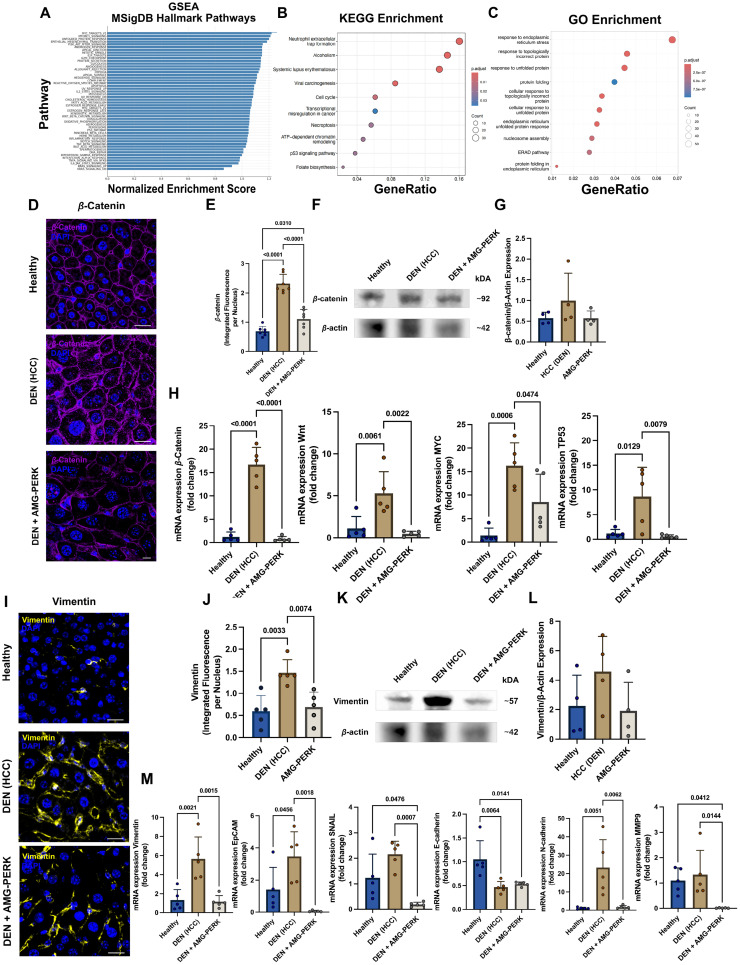

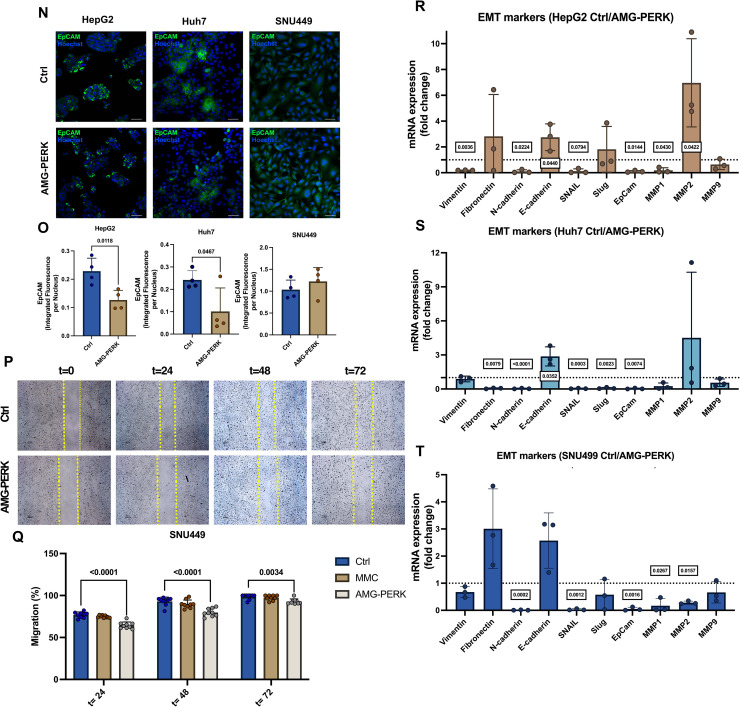
ER-stress induction and PERK inhibition modulate oncogenic signaling and epithelial–mesenchymal transition (EMT). (A) Gene Set Enrichment Analysis (GSEA) of tunicamycin-treated HepG2 cells using MSigDB Hallmark gene sets, showing enrichment of UPR, EMT, inflammatory, and MYC-related pathways. (B) KEGG pathway enrichment analysis highlights transcriptional responses involving immune signaling, carcinogenesis, and cell cycle regulation. (C) Gene Ontology (GO) enrichment analysis identifying ER-stress-related biological processes. (D) Representative β-catenin immunofluorescence images in healthy, DEN-induced, and DEN + AMG-PERK livers. Scale bar: 20 μm; N = 5 mice per group. (E) Quantification of the number of β-catenin positive cells, normalized to nuclear staining (DAPI). (F) Representative western blot of β-catenin and β-actin as loading control in healthy liver, DEN-induced HCC and DEN-induced HCC treated with AMG-PERK. (G) Quantification of β-catenin, normalized to β-actin. (H) qPCR analysis of β-catenin, Wnt2, MYC, and TP53 mRNA expression. N = 5 mice per group (I) Representative vimentin immunofluorescence images from healthy, DEN-induced, and DEN + AMG-PERK livers. Scale bar: 20 μm; N = 5 mice per group. (J) Quantification of the number of vimentin positive cells normalized to nuclear staining (DAPI). (K) Representative western blot of Vimentin and β-actin as loading control in healthy liver, DEN-induced HCC and DEN-induced HCC treated with AMG-PERK. (L) Quantification of vimentin, normalized to β-actin. (M) qPCR analysis of EMT-related markers (vimentin, EpCAM, SNAIL, E-cadherin, N-cadherin, MMP9). N = 5 mice per group. (N) Representative EpCAM immunofluorescence images in HepG2, Huh7, and SNU449 cells treated with AMG-PERK. Scale bar: 20 μm; N = 4 biological replicates and 3 technical replicates. (O) Quantification of EpCAM immunofluorescence normalized to nucleus (DAPI). (P) Scratch wound on SNU449 cells treated with AMG-PERK with the presence of mitomycin C or control. (Q) Quantification of wound-closure (%) in SNU449 cells at 24, 48, and 72 hours following scratch injury, comparing AMG-PERK treatment to control (presented as percentage of wound area closure). N = 3 biological replicates and 3 technical replicates per group, scale bars represent 100 μm. (R-T) qPCR analysis of EMT-related genes in HepG2 (R), Huh7 (S), and SNU449 (T) following AMG-PERK treatment. Expression values normalized to control (set to 1). Statistical significance indicated by boxed p-values. Bars represent mean with ± SD. N = 3 biological replicates per group.

To assess how ER-stress influences transcriptional programs associated with tumor progression, we analyzed publicly available RNA-sequencing data (GSE208391) from HepG2 cells treated with the ER-stress inducer tunicamycin. GSEA confirmed upregulation of the UPR (NES = 1.20, adj. p = 0.099), alongside enrichment of epithelial-to-mesenchymal transition (EMT) and inflammatory signaling (Fig. 5A). Enrichment of MYC target genes (NES = 1.26, adj. p = 0.049) and apical junction pathways further supports a stress-driven shift toward a pro-tumorigenic state. Complementary analyses using KEGG and GO corroborated these findings, identifying overrepresented pathways related to DNA damage, immune activation, protein folding, and interleukin signaling (Fig. 5B, C). Although several individual p-values were modest, the consistent enrichment across independent databases suggests that ER-stress activation is associated with transcriptional programs relevant to tumor plasticity, inflammation, and stromal interactions.

To complement these transcriptomic observations, we investigated whether inhibiting ER-stress with AMG-PERK modulates oncogenic signaling, EMT markers, or motility *in vivo* and *in vitro*. Immunofluorescence staining of liver tissues from DEN-induced HCC mice showed increased β-catenin expression, which was substantially reduced following AMG-PERK treatment (Fig. 5D, E). Western blot analysis confirmed reduced β-catenin protein levels, and qPCR analysis showed reduced mRNA levels of β-catenin, MYC, Wnt2, and TP53 in AMG-PERK-treated mice (Fig. 5F–H). Immunofluorescence staining of liver tissues showed increased vimentin expression in DEN-induced tumors, which was reduced following AMG-PERK treatment (Fig. 5I, J). Western blot analysis confirmed this decrease in vimentin protein levels (Fig. 5K, L). Further qPCR analysis of EMT-associated genes revealed a heterogeneous pattern. Vimentin and EpCAM were both upregulated in DEN-induced tumors and decreased after AMG-PERK treatment. N-cadherin showed a marked increase in DEN livers that was substantially reduced by AMG-PERK. E-cadherin expression decreased in DEN-induced tumors but was largely unchanged by AMG-PERK treatment. SNAIL expression showed a modest increase in DEN livers with partial reduction after AMG-PERK; and MMP9 levels were similar between healthy and DEN-induced livers but decreased following AMG-PERK exposure (Fig. 5M).

Similar heterogenous trends were observed in HCC cell lines (HepG2, Huh7, and SNU449) following AMG-PERK exposure (Fig. 5R, T). Immunofluorescence staining showed high EpCAM expression in untreated HCC cell lines, which was significantly reduced by AMG-PERK in HepG2 (P = 0.0118) and Huh7 (P = 0.0467) cells, but not in SNU449 (Fig. 5N, O). To assess whether the observed transcriptional changes translate into altered tumor cell behavior, we next examined the effects of PERK inhibition on cell migration. In a wound healing assay, SNU449 cells treated with AMG-PERK displayed significantly slower wound closure at 24, 48, and 72 hours compared with controls (Fig. 5P, Q). We then analyzed EMT-related gene expression in three HCC cell lines. In HepG2 cells, AMG PERK decreased the expression of several mesenchymal or migratory markers, including vimentin, N-cadherin, SNAIL, EPCAM, MMP1, and MMP9, while increasing levels of fibronectin, E-cadherin, SLUG, and MMP2 (Fig. 5R). In Huh7 cells, AMG PERK reduced fibronectin, N-cadherin, SNAIL, SLUG, EPCAM, MMP1, and MMP9, while E-cadherin expression increased (Fig. 5S). In SNU449 cells, AMG-PERK decreased N-cadherin, SNAIL, EPCAM, MMP1, and MMP2, with a concomitant increase in E-cadherin (Fig. 5T). Together, these data show that ER-stress modulation influences multiple oncogenic and EMT-associated transcriptional programs, although the magnitude and direction of these changes vary across cellular contexts.

## Discussion

Endoplasmic reticulum (ER) stress is increasingly recognized as a hallmark of tumor biology, contributing to cell survival under adverse conditions, such as hypoxia, nutrient deprivation, and oxidative stress. Activation of the unfolded protein response (UPR) enables cancer cells to adapt to these stresses by modulating cytokine secretion, growth factor signaling, and immune evasion [22,55]. Among the UPR sensors, the PERK pathway contributes to tumor growth, influencing apoptotic signaling, autophagy, DNA repair, as well as drug response [24,56,57]. In this study, we examined the contribution of PERK signaling to hepatocellular carcinoma (HCC) development and microenvironmental remodeling, with a particular focus on early carcinogenesis.

Pharmacologic inhibition of PERK with AMG-PERK effectively suppressed ER-stress markers both *in vitro* and *in vivo* and reduced tumor burden, proliferation, and cell viability. These results align with previous studies, showing that PERK and its downstream signaling pathways modulate the activity of the p53 protein, highlighting their potential as early prognostic biomarkers in lung cancer [58]. Mechanistic studies in other malignancies, such as glioblastoma, have found that simultaneous inhibition of EZH2 and CDK4/6 can alter ER-mitochondrial homeostasis and enhance cancer cell sensitivity, supporting the potential of pharmacologic ER-stress modulation to improve treatment efficacy [59]. PERK has also been shown to play a role in the formation of malignant lesions in breast cancer [60] and in the survival of quiescent cancer cells [61].

Beyond tumor-intrinsic adaptations, our data indicate that ER-stress modulation also influences stromal activation. Fibrosis and inflammatory signaling were reduced in DEN-induced HCC livers following PERK inhibition, accompanied by decreased collagen deposition, lower αSMA expression, and reduced pro-inflammatory cytokine levels. These observations are consistent with previous studies linking chronic ER-stress and CHOP activation to inflammation and fibrosis [62,63], and with broader evidence that the UPR contributes to shaping a pro-tumorigenic microenvironment [64,65]. Notably, the reduction in inflammatory gene expression in PERK-inhibited mice extended slightly below levels observed in healthy controls. This likely reflects suppression of baseline, age-associated low-grade hepatic inflammation, as mice at the experimental endpoint (∼7 months of age) are considered mature adults, rather than young animals. Age-associated increases in inflammatory signaling have been reported to precede overt fibrosis, with TNFα identified as a key driver of gradual hepatic inflammatory changes during aging [66]. Importantly, this low-grade inflammation in control mice was not accompanied by measurable fibrosis, but may represent a clinically relevant baseline, given that HCC typically arises in older patients. Although our findings support a role for ER-stress signaling in HSC activation *in vitro*, additional work will be needed to determine which components of the UPR, and which tumor-derived factors, are responsible for these effects *in vivo*. In this regard, Zhou et al. [67] recently demonstrated that hepatocyte-derived signals can support stellate cell activation and fibrogenesis in HCC through paracrine crosstalk, further highlighting the regulating role of tumor secreted factors in microenvironmental remodeling.

In exploring potential mediators of tumor-stroma communication, we assessed GP73, a secreted glycoprotein known to be induced by ER-stress conditions and associated with HCC progression [29]. ER-stress has been shown to enhance the secretion of GP73 from HCC cells, which can then bind to glucose-regulated protein 78 (GRP78) on neighboring immune cells, particularly macrophages, thereby propagating ER-stress signals and promoting a TAM phenotype that supports tumor progression [29,68]. *In vivo*, high GP73 and CD206 expressions correlate with poor patient outcomes, while GP73 inhibition reduces TAM density, suppresses tumor growth, and prolongs survival [29]. Moreover, GP73 has been shown to enhance the malignant phenotype of HCC cells. It facilitates the secretion of alpha-fetoprotein (AFP) and, independently, promotes proliferation and metastasis of HCC cells [69]. Furthermore, GP73′s role extends to modulating immune responses, as it has been shown to influence the function of cytotoxic T cells by regulating HIF-1α and glycolysis pathways [70]. Based on these findings, we demonstrate GP73 expression was elevated in malignant hepatocytes in scRNA-seq and patient datasets, and PERK inhibition reduced GP73 expression *in vivo*. In HCC cell lines, ER-stress induction increased GP73 secretion, whereas co-treatment with AMG-PERK attenuated this response. While these observations are consistent with GP73 being ER-stress responsive, they do not establish a direct mechanistic link between PERK activation and GP73 regulation in tumors; however, they support the idea that GP73 levels reflect ER-stress activity within malignant hepatocytes.

Functional assays revealed that GP73 can activate hepatic stellate cells (HSCs) *in vitro*, increasing ER-stress markers and fibrotic gene expression. These responses were reduced by a GRP78-blocking antibody targeting its extracellular N-terminus [29], indicating that GP73 can signal through GRP78 in HSCs under experimental conditions. Tumor-conditioned medium produced similar effects and was also sensitive to GRP78 blockade. While these results highlight a potential mechanism through which GP73 could contribute to stromal activation, they do not exclude the involvement of additional secreted mediators. GRP78 is a promiscuous cell-surface receptor that engages a broad repertoire of tumor-secreted ligands, including secreted GRP78 itself, activated α2-macroglobulin, Par-4, Kringle-5, and others [[71], [72], [73], [74]]. Solid tumors also secrete GRP78, which can act in an autocrine or paracrine manner via cell surface GRP78 to drive stromal activation, immune modulation, and mesenchymal differentiation [73,74]. Therefore, while our data indicate that GP73 is *capable* of signaling through GRP78 in HSCs, they do not exclude the possibility that additional GRP78-binding factors within tumor-conditioned medium contribute to the observed effects. Further work will be required to isolate the relative contribution of GP73 among the broader set of GRP78-dependent mediators. Also, while our findings indicate that GP73 can activate stellate cells *in vitro* and that both PERK inhibition and GRP78 blockade attenuate stromal activation, the current study does not establish whether GP73 or HSC activation is necessary for tumor progression *in vivo*. Further loss-of-function studies such as GOLM1 knockdown in tumor cells or stellate-specific GRP78 or perk deletion will be required to define the causal contribution of this pathway to hepatocarcinogenesis. In addition, the *in vitro* experiments in our study were performed using LX-2 cells, an immortalized human hepatic stellate cell line that exhibits a stable, activated myofibroblast-like phenotype. While LX-2 cells are a well-validated and widely used model for mechanistic studies of activated HSC signaling, they do not recapitulate the quiescent stellate cell state, and future validation in primary human HSCs or more complex liver models will be important [75,76].

GRP78 signaling in HSCs may also influence the hepatic immune microenvironment. Prior work has shown that GRP78 knockdown in primary activated HSCs reduces secretion of pro-inflammatory cytokines such as IL-1β and TNFα, suggesting a role for HSC-intrinsic GRP78 signaling in shaping inflammatory cues within the liver [77]. In addition, ER-stress pathways intersect with TGF-β signaling, a central regulator of both fibrosis and immune modulation, and TGF-β-mediated activation of HSCs has been linked to ER-phagy and ER homeostasis remodeling [78,79]. Furthermore, GP73 could have also directly influenced TGF-beta signaling [80]. Together, these observations raise the possibility that GRP78-dependent signaling in HSCs indirectly modulates immune cell activation and inflammatory tone in the tumor microenvironment, an important question for future investigation.

At the tumor-cell level, our data indicate that ER-stress modulation influences transcriptional programs associated with oncogenic signaling and epithelial-mesenchymal transition (EMT), both of which are well established drivers of HCC progression [81]. Transcriptomic analysis of tunicamycin-treated HepG2 cells showed enrichment of EMT, inflammatory, and MYC-related pathways alongside UPR activation, emphasizing transcriptional programs associated with aggressive HCC phenotypes, molecular heterogeneity, and immune microenvironmental alterations [82,83]. Consistent with these observations, PERK inhibition reduced β-catenin and MYC expression in DEN-induced tumors and attenuated several mesenchymal markers, including vimentin, N-cadherin, and SNAIL, *in vivo* and across HCC cell lines. Other EMT-associated genes displayed a heterogeneous pattern: MMP9 levels were unchanged in DEN-induced tumors but decreased with AMG-PERK, while E-cadherin was downregulated in DEN-treated mice and remained largely unchanged after treatment. In HCC cell lines, PERK inhibition reduced EpCAM and additional EMT-related genes in a cell-line-specific manner. Functionally, AMG-PERK treatment impaired cell migration in wound-healing assays, consistent with an overall shift toward a less mesenchymal phenotype. These findings support a link between ER-stress signaling and EMT-associated transcriptional programs, in line with previous studies [[84], [85], [86]], but also highlight that ER-stress-dependent EMT regulation is heterogeneous and may depend on cellular context. Further studies are needed to determine whether ER-stress activation is sufficient to drive EMT.

Finally, our results show that ER-stress promotes proliferative signaling, as supported by increased PCNA and Ki67 expression in DEN-induced tumors, both of which were suppressed by AMG-PERK. This supports the broader view that PERK signaling sustains or promotes cancer cell proliferation, in line with previous findings [[87], [88], [89]].

Taken together, our findings show that ER-stress signaling, particularly through PERK, contributes to multiple features of early hepatocarcinogenesis, including tumor growth, stromal activation, inflammatory signaling, and transcriptional programs associated with EMT and proliferation. GP73 emerges as an ER-stress mediated secreted factor capable of activating stellate cells. Importantly, pharmacologic inhibition of PERK during early carcinogenesis affected both tumor-intrinsic and microenvironmental processes, supporting PERK-targeted therapy as a potential preventive treatment strategy in cirrhotic patients at risk for developing HCC.

## Data availability

The images shown in 3a-b are derived from the Single-Cell Atlas in liver cancer: https://scatlaslc.ccr.cancer.gov/. The results shown in Fig. 3C here are based on data generated by the TCGA Research Network: https://www.cancer.gov/tcga. The images shown in Fig. 3d, e are obtained from the human protein atlas: https://www.proteinatlas.org. The following GEO datasets were included: liver-specific PERK knockout mice (LsPERK-KO; accession: GSE29929 [48]), PERK knock out LX2 stellate (GSE292002) [49], and PERK-deficient bone marrow-derived macrophages (Accession: GSE165836) [26].

## CRediT authorship contribution statement

**Jaafar Khaled:** Writing – review & editing, Writing – original draft, Visualization, Validation, Methodology, Investigation, Formal analysis. **Maria Kopsida:** Writing – review & editing, Writing – original draft, Methodology, Investigation, Formal analysis. **Tania Payo Serafín:** Writing – review & editing, Methodology, Investigation. **Sofi Sennefelt Nyman:** Writing – review & editing, Methodology, Investigation. **Fredrik Rorsman:** Writing – review & editing, Supervision, Funding acquisition. **Charlotte Ebeling Barbier:** Writing – review & editing, Supervision, Funding acquisition, Formal analysis. **Hans Lennernäs:** Writing – review & editing, Supervision, Funding acquisition, Conceptualization. **Markus Sjöblom:** Writing – review & editing, Supervision, Project administration, Methodology. **Femke Heindryckx:** Writing – review & editing, Writing – original draft, Visualization, Validation, Supervision, Resources, Project administration, Methodology, Investigation, Funding acquisition, Formal analysis, Conceptualization.

## Declaration of competing interest

The authors declare no competing interest. This research was funded by The Swedish Cancer Foundation (Cancerfonden), The Swedish Society for Medical Research (SSMF), The Swedish Research Council, and the Göran Gustafsson foundation. The funders had no role in the design of the study, collection and analysis of data and decision to publish.

## Appendix Supplementary materials

Image, application 1
