# Supplementary material for: Inhibition of PERK signaling suppresses tumor progression and blocks GP73-GRP78-dependent stromal activation in hepatocellular carcinoma
Source: Neoplasia. 2026 Jun 16;79:101329. doi: 10.1016/j.neo.2026.101329 (PMC13292247; doi:10.1016/j.neo.2026.101329)
Supplement: Supplementary file 1 [file mmc1.docx]

**Supplementary figures**


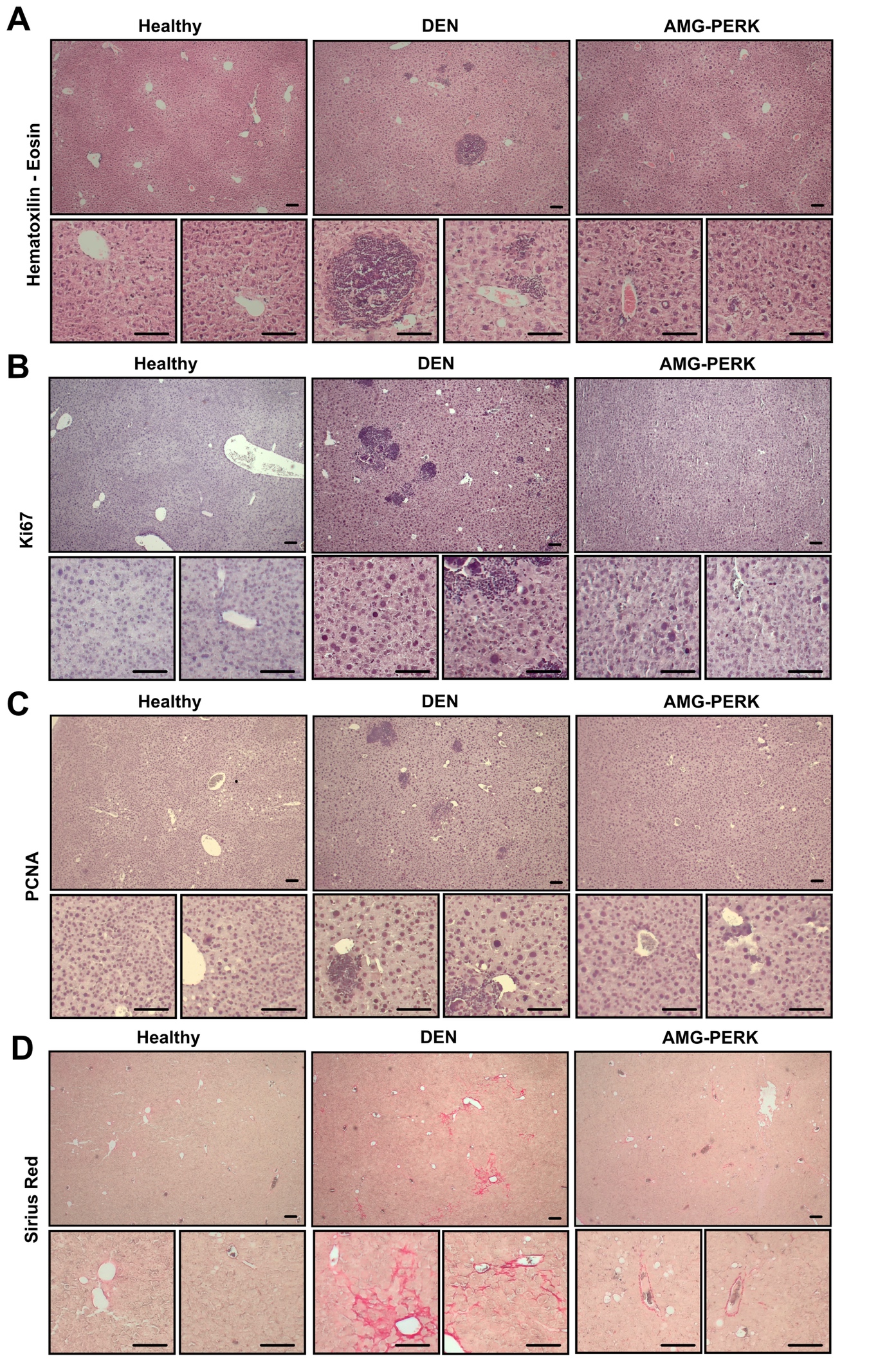


**Supplementary figure S1: Overview images acquired at 5x (H&E, Sirius Red, Ki67, and PCNA).** Scale bars represent 100 μm

**
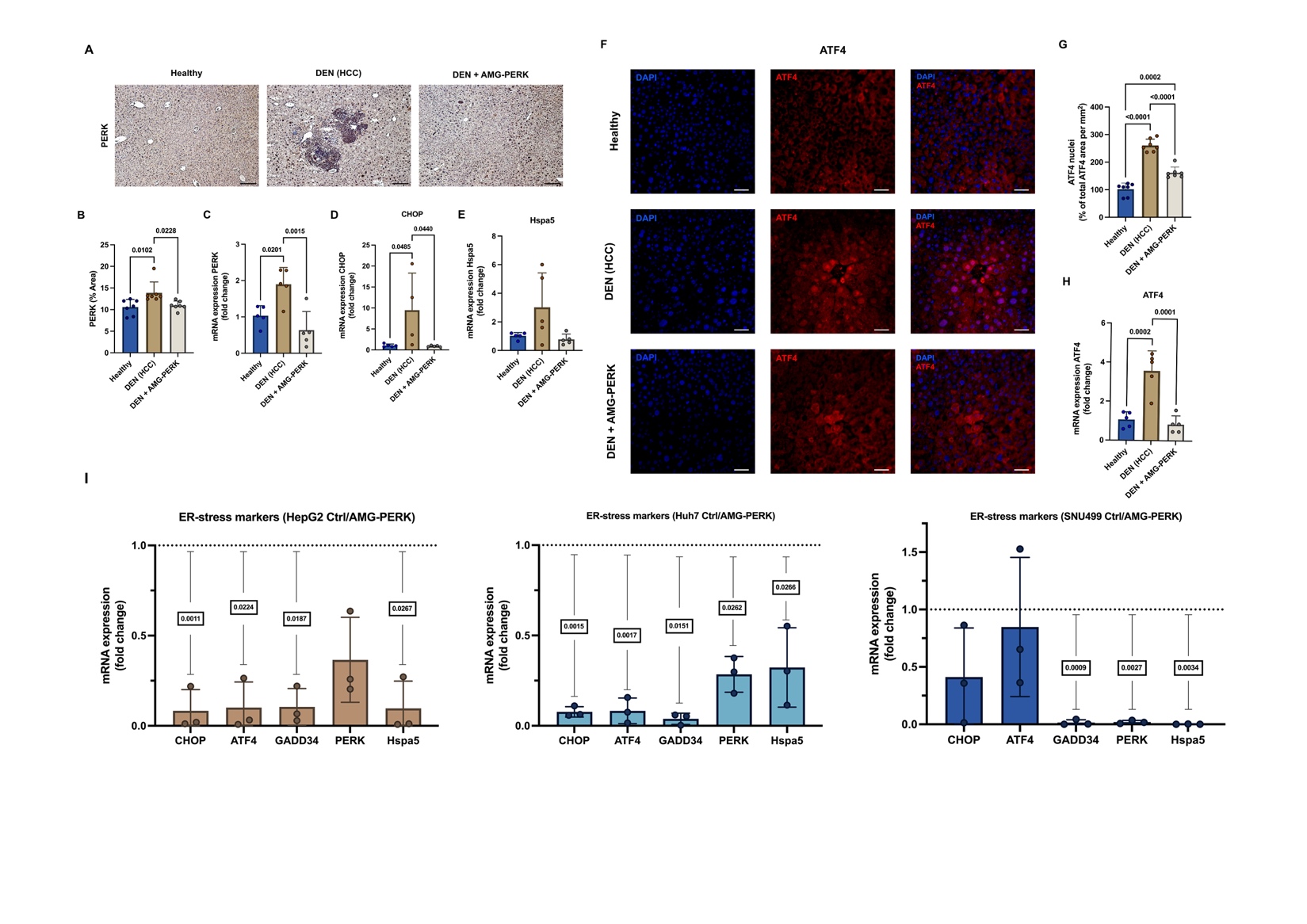
**

**Supplementary figure S2: Treatment with AMG-PERK modifies the expression of ER-stress markers.**

**(A)** Representative images of immunohistochemical staining using antibody targeting PERK. **(B)** Quantification of the number of percentage of PERK positive staining per area **(C)** mRNA-expression of ER-stress marker PERK, **(D)** CHOP and **(E)** Hspa5. **(F)** Representative images of liver tissue sections stained with antibodies against ATF4. **(G)** Quantification of ATF4 nuclear localization expressed as the percentage of nuclear ATF4-positive area relative to total ATF4-positive area, normalized to the image area (0.180 mm²). **(H)** mRNA-expression of ER-stress marker ATF4. **(I)** mRNA expression of ER-stress markers CHOP, ATF4, GADD34, PERK and Hsp5 in HepG2, Huh7 and SNU449 treated with AMG-PERK. Expression levels are normalized to the control condition (dashed line, set to 1). Statistical significance is indicated by boxed p-values. Bars represent mean with ± SD. N= 7 mice per group, scale bars represent 100 μm for PERK (A), N= 7 mice per group, scale bars represent 20 μm for ATF4 (D), qPCR N= 5 mice per group. N= 3 biological replicates per group for *in vitro* qPCR.


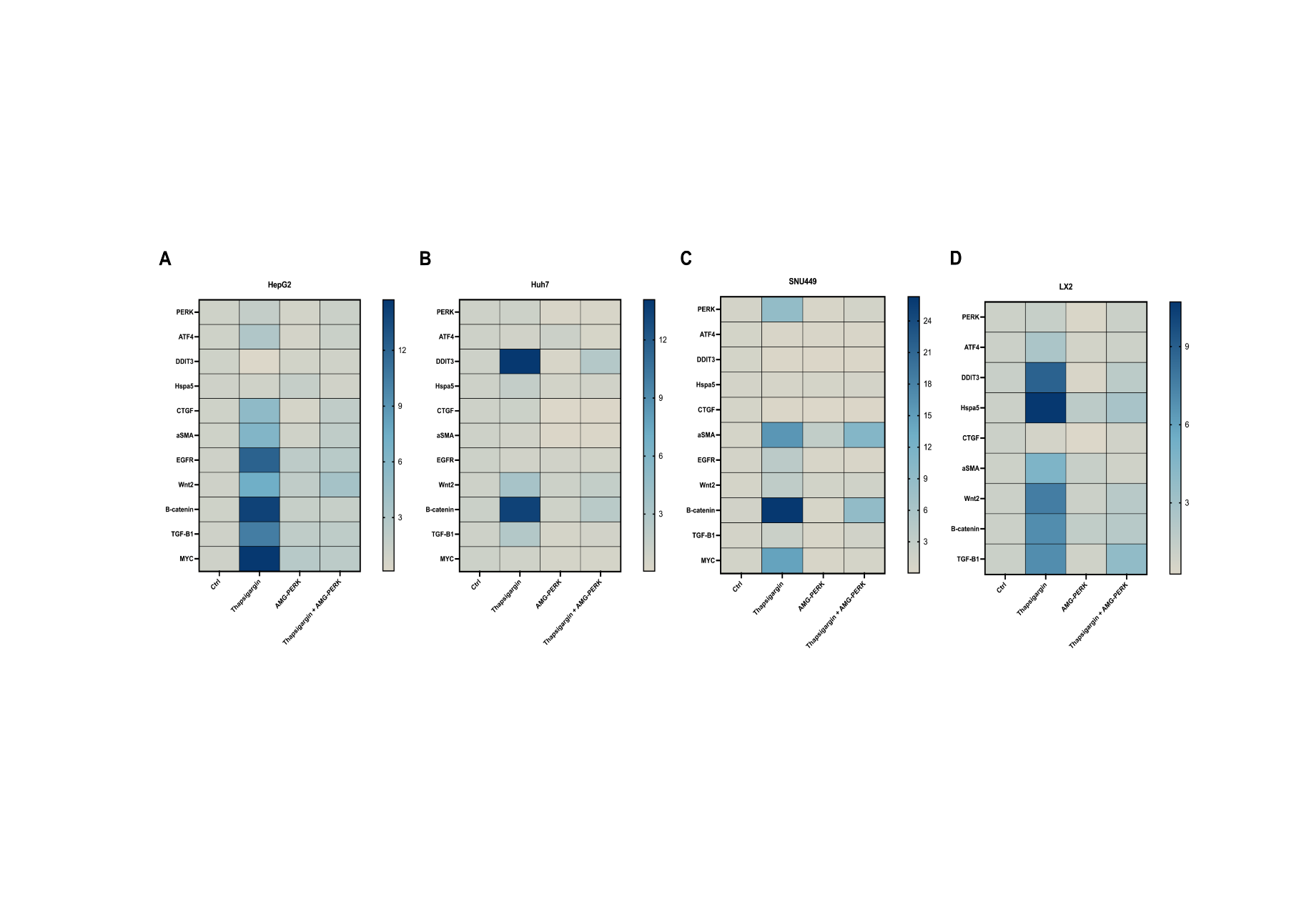


**Supplementary figure S3: Treatment with AMG-PERK and/or thapsigargin alters the expression of ER-stress, fibrotic and oncogenic markers *in vitro*.**

Heatmaps showing fold change mRNA expression of ER-stress, fibrotic and oncogenic markers in **(A)** HepG2 treated with AMG-PERK and/or thapsigargin, **(B)** Huh7, **(C)** SNU449 and **(D)** LX-2. N= 3 biological replicates per group for *in vitro* qPCR.


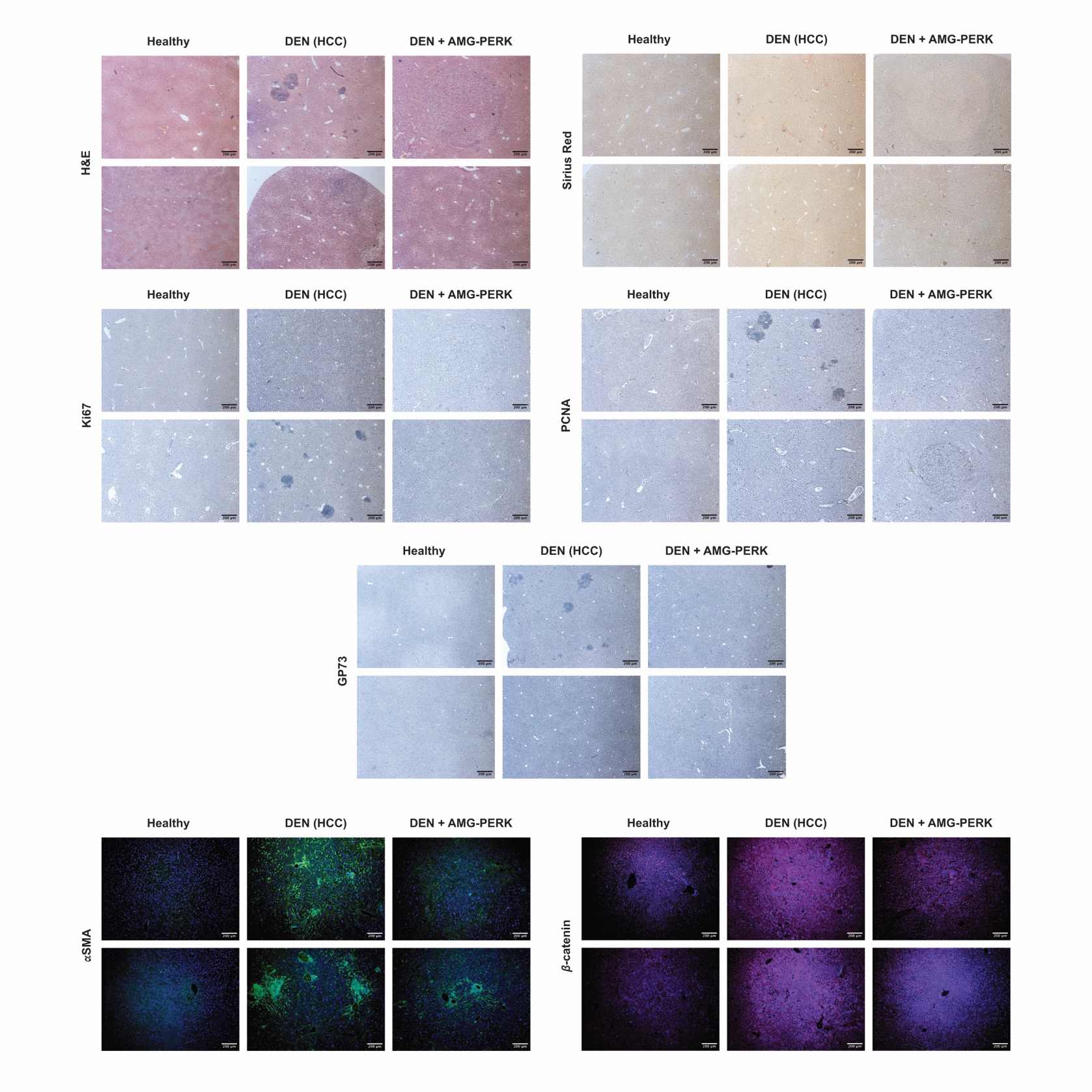


**Supplementary figure S4: Overview images acquired of 𝛼SMA and 𝛽-catenin.** Scale bars represent 100 μm


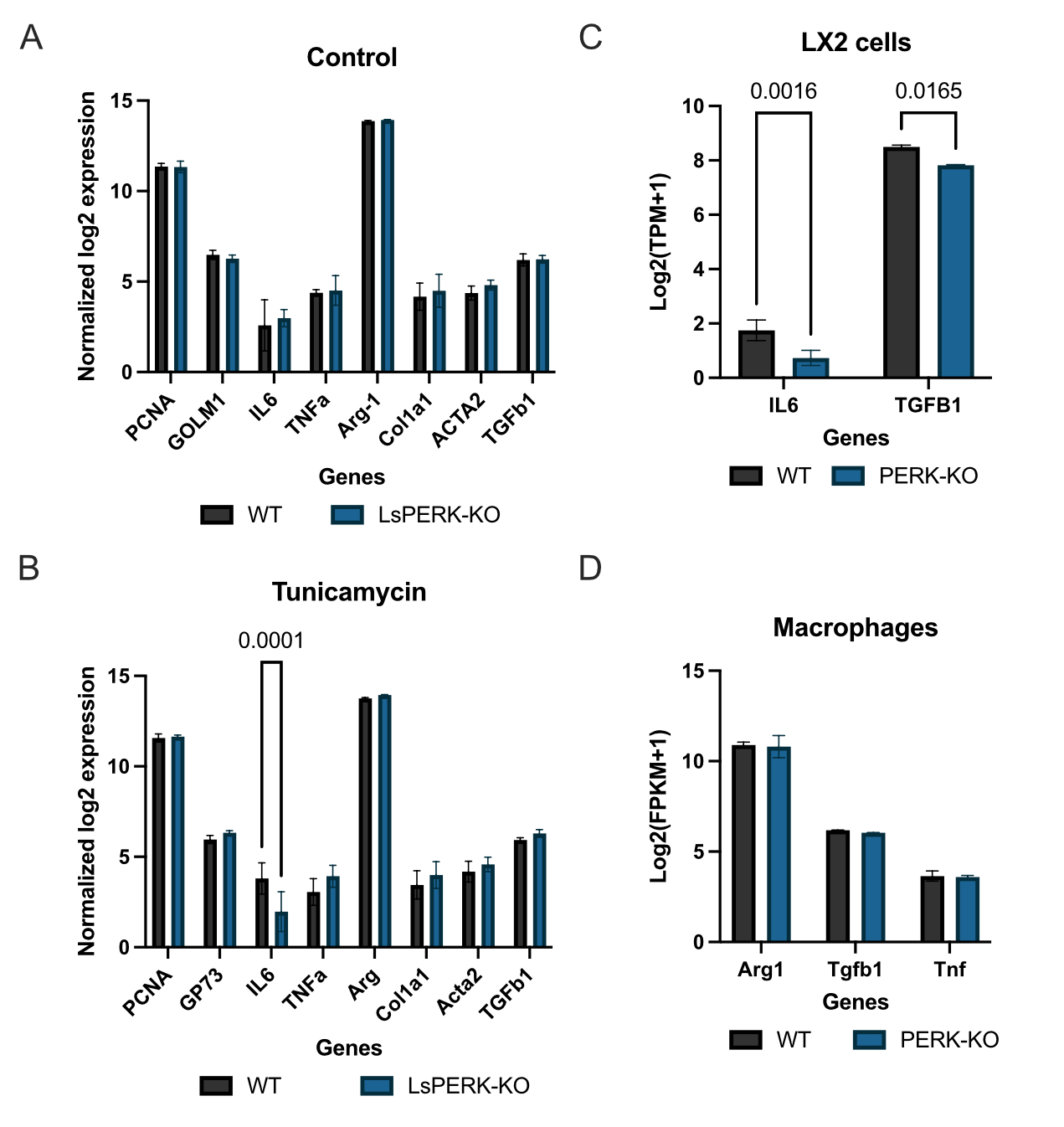


**Supplementary figure S5: Effects of genetic PERK depletion on inflammatory and fibrogenic responses under stress conditions.**

**(A)** Basal expression of inflammatory and fibrogenic markers (TNFα, Arg-1, Col1a1, ACTA2, and TGFβ1) in liver-specific PERK knockout (LsPERK-KO) mice compared to wild type (WT) controls. **(B)** Expression of inflammatory and fibrogenic markers (TNFα, Arg-1, Col1a1, ACTA2, and TGFβ1) in WT and LsPERK-KO mice following exposure to ER stress inducer tunicamycin. **(C)** Expression of IL6 and TGFβ1 in PERK-deficient LX-2 cells exposed to palmitate-induced lipotoxic stress. **(D)** Expression of Arg-1, TGFβ1, and TNFα in PERK-deficient bone marrow-derived macrophages. Data were extracted and analyzed from publicly available datasets (GSE29929, GSE292002 and GSE165836).


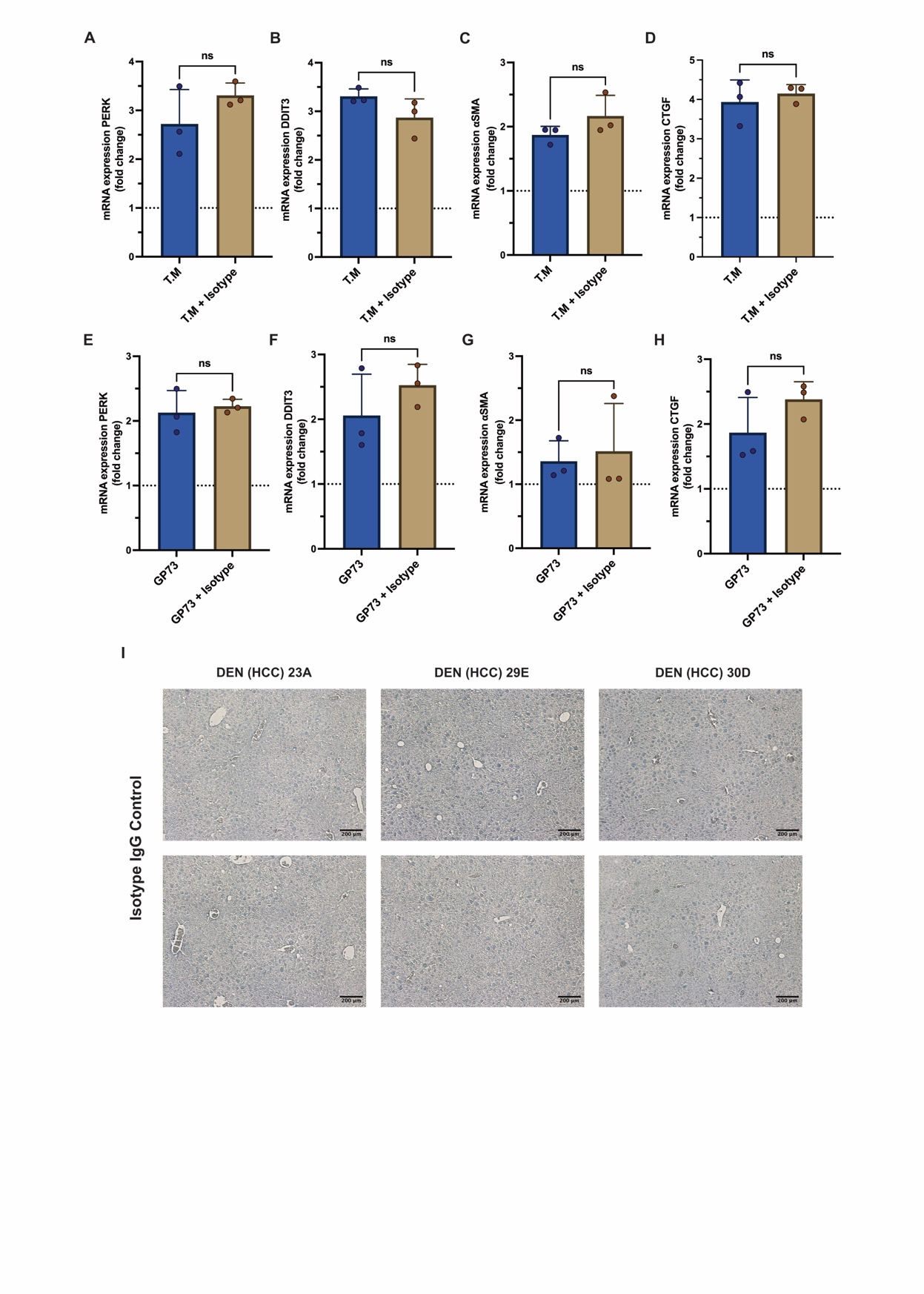


**Supplementary figure S6: Isotype control validation of GRP78 antibody treatment in LX-2 cells.**

LX-2 cells were treated with control medium, tumor-conditioned medium derived from Huh7 cells (T.M.), recombinant GP73, T.M. + isotype IgG control, or GP73 + isotype IgG control. mRNA expression of **(A)** PERK, **(B)** DDIT3, **(C)** αSMA, and **(D)** CTGF in LX 2 cells treated with T.M. or T.M + isotype IgG control. mRNA expression of **(E)** PERK, **(F)** DDIT3, **(G)** αSMA, and **(H)** CTGF in LX 2 cells treated with GP73 or GP73 + isotype IgG control. **(I)** Representative images of immunohistochemical staining using IgG isotype control on 3 different DEN-induced HCC mice tissue sections.
